# The isolation and expression analysis of cinnamate 4-hydroxylase and chalcone synthase genes of *Scrophularia striata* under different abiotic elicitors

**DOI:** 10.1038/s41598-022-12361-8

**Published:** 2022-05-17

**Authors:** Zeinab Rostami, Arash Fazeli, Zohreh Hojati

**Affiliations:** 1 Plant Breeding, Faculty of Agriculture, University of Ilam, Ilam, Iran; 2 Department of Agronomy and Plant Breeding, Faculty of Agriculture, University of Ilam, Ilam, Iran; 3 Department of Cellular and Molecular Biology and Microbiology, Faculty of Biological Science and Technologies, University of Isfahan, Isfahan, Iran

**Keywords:** Molecular biology, Physiology, Plant sciences

## Abstract

The phenylpropanoid pathway serves as a rich source of metabolites in plants, and it is considered as a starting point for the production of many other important compounds such as the flavonoids, flavonols, coumarins, and lignans. *Scrophularia striata* is a member of the Lamiaceae family with some biological activities similar to flavonoid compounds such as antioxidant, antibacterial, anti-inflammatory and analgesic activities. Cinnamate 4-hydroxylase (C4H) and Chalcone synthase (CHS) are key enzymes of the phenylpropanoid pathway, leading to the biosynthesis of several secondary metabolites. In this study, two *S. striata* CHS and C4H were isolated and then analyzed. The investigation of the expression of these genes was performed under the effects of three salicylic acid (SA), jasmonic acid (JA), and gibberellic acid (GA) at concentrations of 100 and 300 ppm with a completely randomized design at the transcript level using Real Time PCR method. These have different expression patterns at developmental stages. Moreover, these genes present different sensitivities to hormonal treatment. Considering the total results, it was found that the amount of expression of these genes during the reproductive phase is higher than that of the vegetative phase. Additionally, the treatment of 300 ppm SA in the reproductive phase is the most effective treatment on increasing the corresponding phenylpropanoid compounds. A correlation analysis was performed between the phenylpropanoid compounds content and both CHS and C4H expression values at different phenological development stages. The results indicate that the expression variations of both CHS and C4H are significantly related to the changes in total phenolic content. We believe that the isolation of CHS and C4H can be helpful in better understanding phenylpropanoid metabolis.

## Introduction

The use of herbs for the prevention and treatment of diseases has gained increasing attention worldwide^1^. *S. striata* Boiss with the local name of Teshnedari, is a medicinal plant of Scropulariacease family, consisting of 220 genera, among which the Scrophularia is known as one of the largest genus. These genera are mainly distributed in mountainous areas (such as *Scrophularia farinosa* Boiss and *Scrophularia amplexicaulis* Benth) and rarely in deserts (*Scrophularia deserti* Delile)^2^. In Iran, this plant is originated from some western and southwestern provinces. For many years, the local inhabitants of these provinces have been using this plant in various forms such as oral decoction, incense, and poultice for the treatment of various diseases such as inflammation, eye and ear infections, skin burns, infectious wounds, episiotomy, pain and digestive disorders, colds, hemorrhoids, and boils^3–6^. The dried roots of Scrophularia species is used as an adjunct for fever, laryngitis, edema, constipation, neuritis, and pharyngitis in Asian medicine^7,8^. According to some findings, phenylpropanoid glycosides and iridoids are the two major secondary metabolites the Scrophularia, showing an obvious therapeutic potential in numerous studies. Several biological effects of phenylpropanoid compounds such as antioxidant, hepatoprotective, anti-tumor, and anti-inflammatory, as well as other beneficial effects have been studied over the past few years^9,10^. Flavonoids have also been reported to have different potentials for the prevention and treatment of many diseases, including viruses^11,12^. Besides, the lack of systematic toxicity and pleiotropic effect of flavonoids has led this compound to be considered as a potential candidate in the treatment of viral diseases, including the treatment of coronavirus^13–16^. The biosynthesis of flavonoids is a complex process involving several enzymatic steps. At the early stages of flavonoid synthesis, phenylalanine derived from shikimate pathway by the enzymes of the phenylpropanoid (including phenylalanine ammonialyase (PAL)), cinnamate 4-hydroxylase (C4H) and C-4-hydroxylase (4CL) was converted to comaryl coenzyme A. Chalcone synthase (CHS), as the first flavonoid synthesizing enzyme, combines comaryl coenzyme A with three molecules of malonyl coenzyme A, in order to produce naringenin chalcone^17^. Next, CHI-mediated naringenin chalcone acted as a precursor molecule for the biosynthesis of pigments anthocyanins, proanthocyanidins, flavonoids, and antimicrobial phytoaxins^18,19^. Many studies have previously introduced cinnamic acid as a channel between PAL and C4H enzymes, which results in the production of p-coumaric acid, acting as an intermediate during the production process of phenylpropanoids^20^. Overall, it has been found that C4H plays a key role in promoting cost-effective production in various plants in the future^21^. CHS, as the gatekeeper of flavonoid biosynthesis, has also been reported to play an important role in pathway’s regulation, and thus in plant defense^22,23^. In another study, Nicholson & Hammerschmidt (1992) reported that CHS could be used as a biochemical marker to evaluate dynamic changes in flavonoid synthesis^24^. Similarly, the results of some previous studies conducted on *Silybum marianum* showed that CHS overexpression is able to increase the production of silymarin and other flavonolignans in capillary roots. Therefore, it offers a new solution to increase silymarin production^25^. Mahon et al. (2021) in their study have reported that the induction of the chalcone synthetase 3 (CHS3) gene in transgenic poplar increased the accumulation of flavonoid naringenin in methanolic extracts of xylem compared to wild-type trees^26^. The CHS gene is widely isolated and characterized from many types of plants^27–31^. Besides genetic manipulations, the production of appropriate secondary metabolites requires the molecules triggering the production, because the biosynthetic pathway of the secondary metabolites is a part of the plant's defense mechanism against pathogens and herbivores. Therefore, it can be said that its expression requires inducing compounds. In general, such compounds with the ability to stimulate the expression of defense pathway genes are called elicitors^32–34^. Some chemical compounds such as salicylic scid (SA), jasmonic acid (JA), and gibberellic acid (GA) were also studied as non-biological elicitors to increase these compounds’ production^35^. In a study, Dao et al. (2011) reported that CHS expression, which is regarded as a part of the plant's growth program, can be induced under some stressful conditions such as UV light, and bacterial or fungal infections. CHS expression also induces the accumulation of flavonoid and isoflavonoid phytoalexins and is involved in the salicylic acid (SA) defense pathway^18^. Ellard-lver and Douglas (1996) in their study have reported that methyl jasmonate is effective on inducing the expression of genes involved in the production of phenylpropanoid compounds in parsley (*Petroselinum crispum*)^36^. The present study was designed in terms of the importance of *S. striata* in traditional medicine among people living in the western and southwestern provinces of Iran. In the present study, the identification and isolation of both CHS and C4H, as the key genes involved in the biosynthetic pathway of flavonoids, were performed. Thereafter, the effect of different concentrations of three elicitors, including salicylic acid (SA), jasmonic acid (JA), and gibberellic acid (GA), on the expression of the above-mentioned genes was investigated at different phenological stages. Considering that there have been few international studies on the marker genes in metabolic pathways in *S. striata* up to now, the present research is the first study investigating different pathways of biosynthesis of valuable compounds in the pharmaceutical and food industries using new genetic engineering methods in the future.

## Results

### Cloning of CHS and C4H Genes

The accession numbers of the two CHS and C4H genes in GenBank are OK288015 and OK288016. CHS and C4H were isolated using PCR, with cDNA from *S. striata* leaves as a template using specific primers (Table 5).

### Expression profiling of *S. striata* CHS and C4H genes affected by various abiotic elicitors at different phenological stages

#### Two-way analysis of variance

The results of two-way ANOVA test for both target genes showed a significant expression difference among the seven treatment groups, regardless of the harvest step (#treat row (first characteristic, P-value < 0.05). As well, a significant expression difference was found between the vegetative and reproductive stages (regardless of the seven treatment groups) (P-value < 0.05 for #stage row (second characteristic). Besides, there was a significant relationship among the fourteen groups in terms of the interaction between the two mentioned characteristics (P-value < 0.05) in the last row. Overall, the results indicate the different expression levels of CHS and C4H genes among the aerial shoots of the plant at vegetative and reproductive growth stages under different hormonal treatments (Table 1).

**Table 1:** The results of two-way analysis of variance for comparing the expression of target genes in the aerial part under different hormonal treatments at different phenological stages.

Source	SS	df	MS	F	P-value
**CHS**					
#Treat	27.82	6	4.63	105.36	< 1*10^−8^
#Stage	12.88	1	12.88	292.66	< 1*10^−8^
Interaction	22.79	6	3.79	86.31	< 1*10^−8^
Error	1.23	28	0.044		
Total	64.73	41			
**C4H**					
#Treat	107.27	6	17.87	463.64	< 1*10^−8^
#Stage	1.97	1	1.97	51.12	< 1*10^−8^
Interaction	30.51	6	5.08	131.89	< 1*10^−8^
Error	1.07	28	0.038		
Total	140.84	41			

Figures showing the expression changes of CHS and C4H genes based on the two variables (hormonal treatment type and harvest time) using two-way analysis of variance.

**Figure 1:**
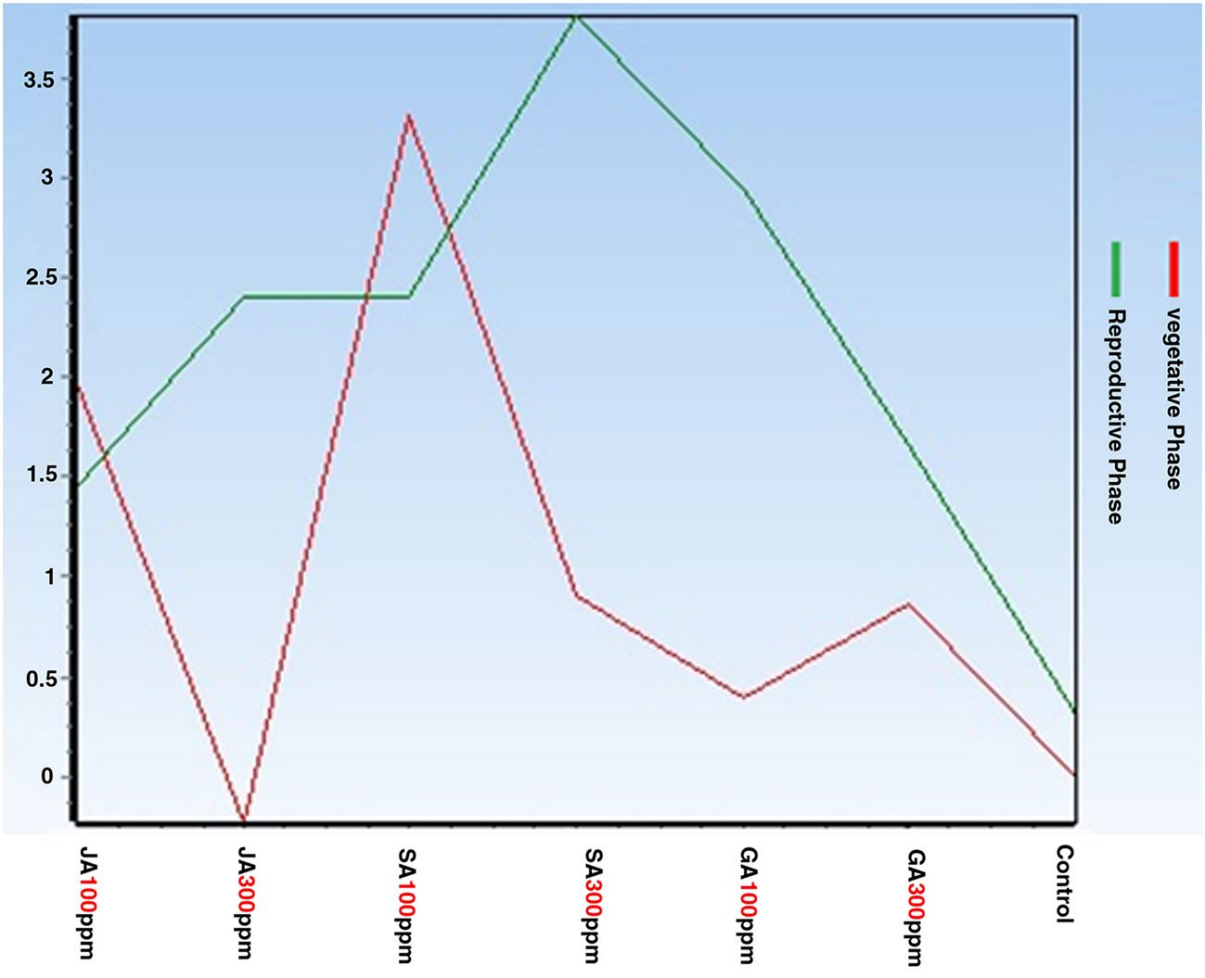
Changes in CHS gene expression in the aerial part under different hormonal treatments at different phenological stages.

**Figure 2:**
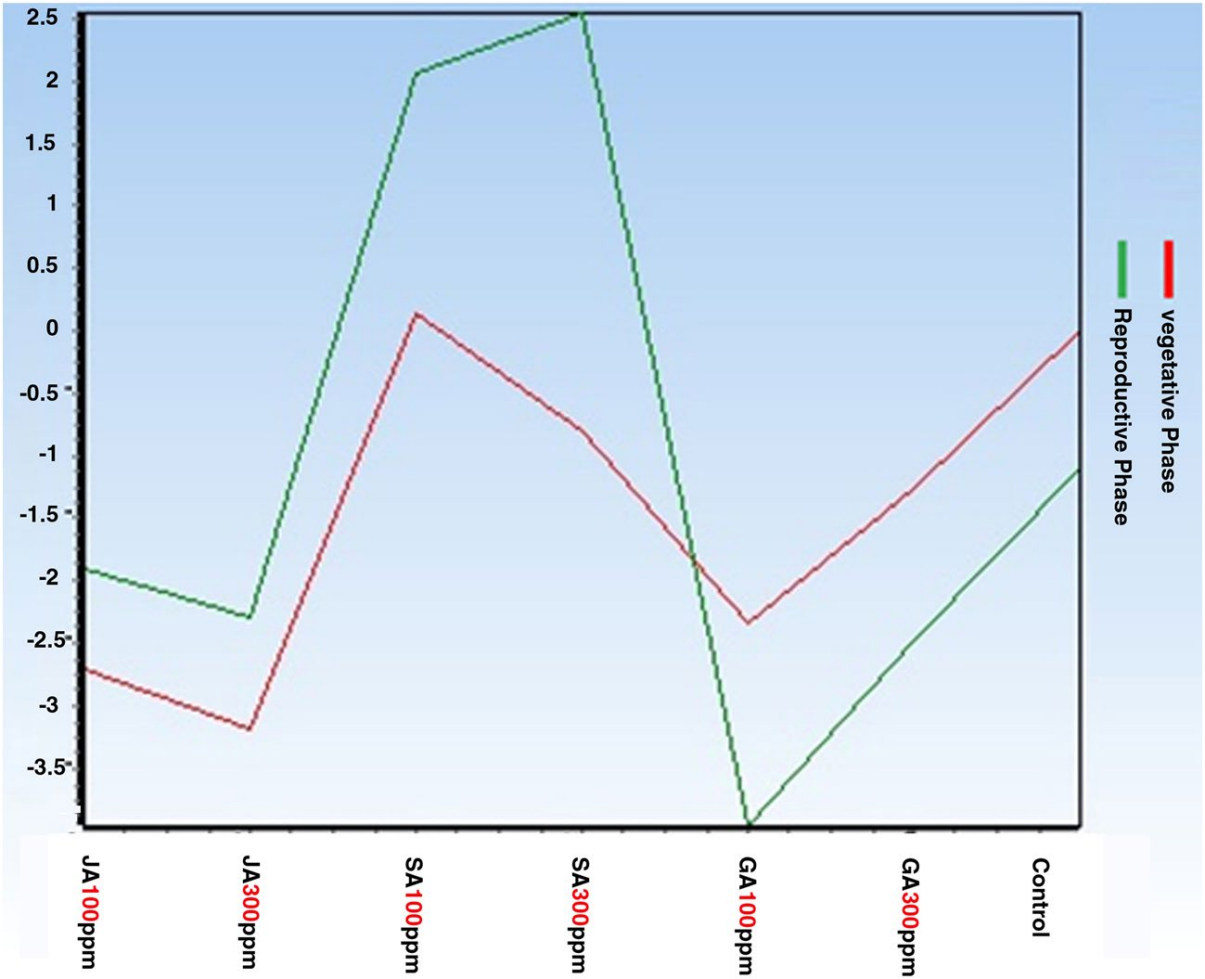
Changes in C4H gene expression in the aerial part under different hormonal treatments at different phenological stages.

Figures 1 and 2 show the expression changes of CHS and C4H target genes based on the two variables. The red and green figures show the changes in the expression of target genes under different experimental treatments in the vegetative and reproductive phases, respectively. According to the figures obtained regarding CHS and C4H target genes, it can be inferred that the expression of these genes using the most experimental treatments in the reproductive phase is higher than that of the vegetative phase, in a way that the gene expression in the SA treatment (300 ppm) has reached its peak level.

#### T-test

**Figure 3:**
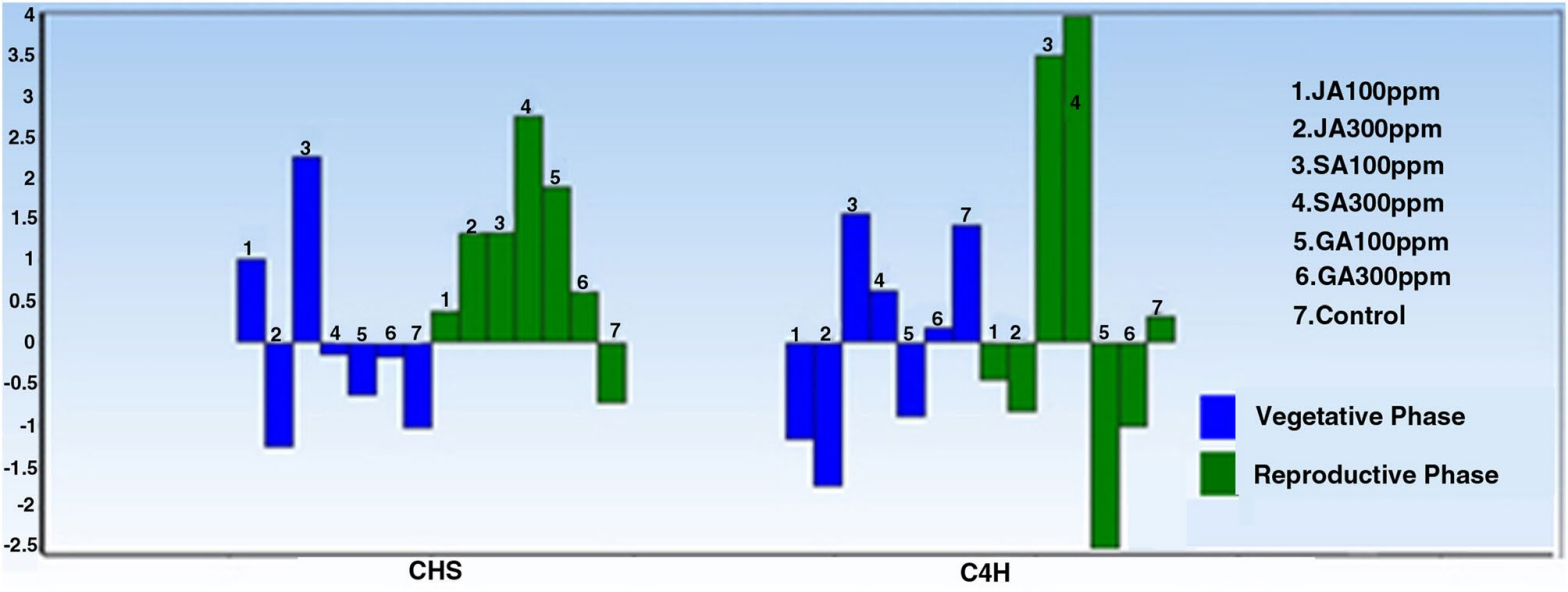
Barplot diagram obtained from T-Test for comparing the expressions of target genes in the aerial part undergone different hormonal treatments at different phenological stages. Accordingly, the diagram obtained for CHS gene shows the increased gene expression in all the treatments performed in the reproductive phase, so that the highest expression level was observed under the SA treatment (300 ppm), and a decrease in the expression was only observed in the reproductive phase in the control group. SA (100 ppm) was found as the most effective treatment on increasing the CHS expression in the vegetative phase. By comparing C4H gene expression diagram in both vegetative and reproductive phases, it was found that different SA concentrations in the reproductive phase have caused a sharp increase in the expression of this gene. Additionally, the diagram obtained for C4H gene shows a decreasing trend under the effect of different concentrations of JA in both vegetative and reproductive phases.

In order to determine the ratio of changes and significance level between the samples harvested from the aerial part in the vegetative and reproductive phases, T-test was performed (Fig. 3, Table 2).

**Table 2:** The results of T-test in terms of estimating the ratio of changes and the significance level between the aerial samples undergone different hormonal treatments at different phenological stages.

Treat	CHS (reproductive)	CHS (vegetative)	C4H (reproductive)	C4H (vegetative)
JA100PPM	0.39491	1.02005	− 0.45172	− 1.18472
JA300PPM	1.35345	− 1.27846	− 0.85131	− 1.75469
SA100PPM	1.35199	2.26979	3.49942	1.5775
SA300PPM	2.76322	− 0.14616	3.98465	0.64315
GA100PPM	1.90158	− 0.64597	− 2.52557	− 0.90613
GA300PPM	0.61411	− 0.17334	− 1.03505	0.18383
Control	− 0.73356	− 1.04591	0.33354	1.44106
Mean	1.09224	0	0.42199	0
Difference (A–B log scale)		1.09224		0.42199
Fold change		2.13205		1.33978

A difference in row (A–B log scale) indicates the -ddct content and row of ratio of the changes also indicates this ratio in target genes in the reproductive phase compared to the vegetative phase. Correspondingly, the ratios of the changes for CHS and C4H genes were obtained as 2.13 and 1.34, respectively.

**Figure 4:**
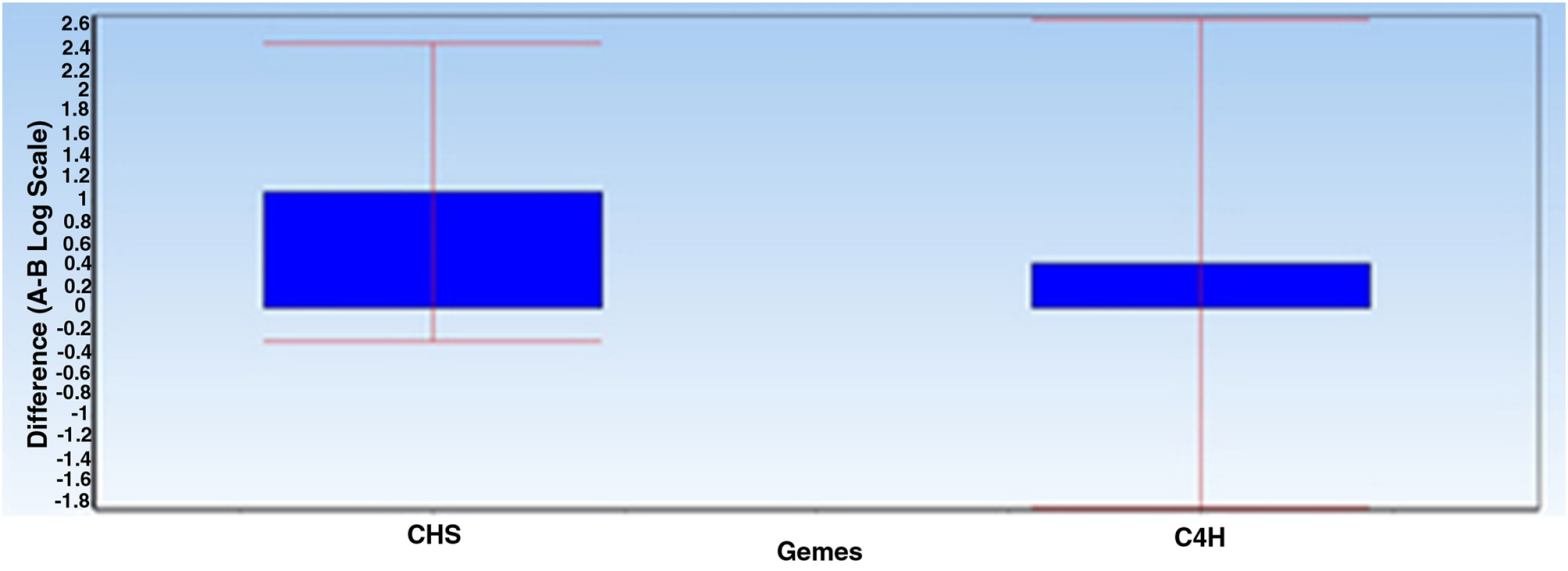
The difference between the mean expressions of target genes in the reproductive phase compared to the vegetative phase. As can be observed, both C4H and CHS target genes overexpressed in the reproductive samples compared to the vegetative samples. However, the overall expression of CHS gene in the reproductive phase was almost twice of that of the vegetative phase. However, in the C4H gene, this overexpression occurred with less intensity (less than 1.5 times).

In addition, Diagram 4 shows the difference between the mean data of the group A (reproductive phase) and group B (vegetative phase) for each gene with error data bar. Since the mean difference between these two groups is -ddCt, the following diagram shows the fold change of the genes in the reproductive group compared to the vegetative group (Fig. 4).

### Changes in the total phenolic, flavonoid, and flavonol contents under the effect of various hormonal treatments at different phenological stages

**Figure 5:**
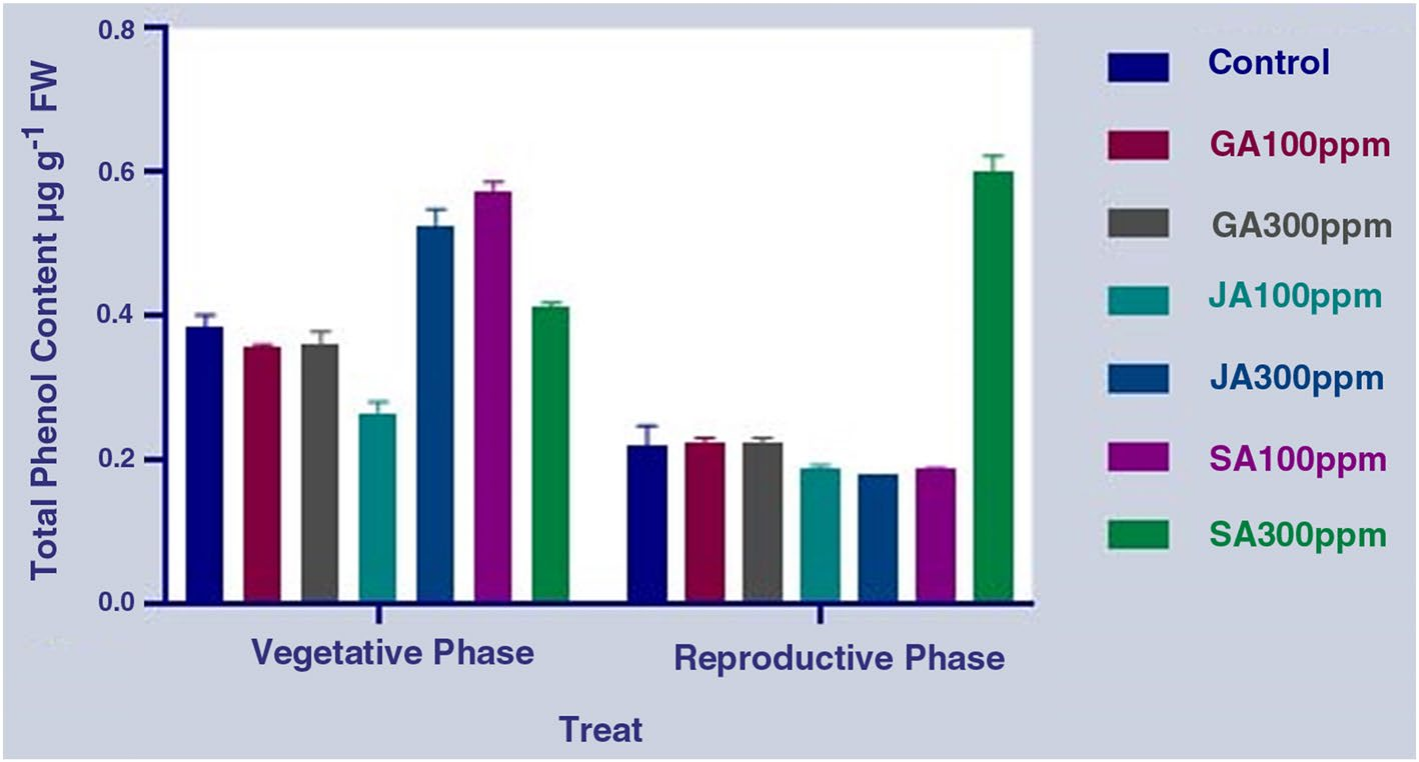
Diagram for comparing the effect of different hormonal treatments on the total phenol content in the aerial part at different phenological stages of *S. striata*.

**Figure 6:**
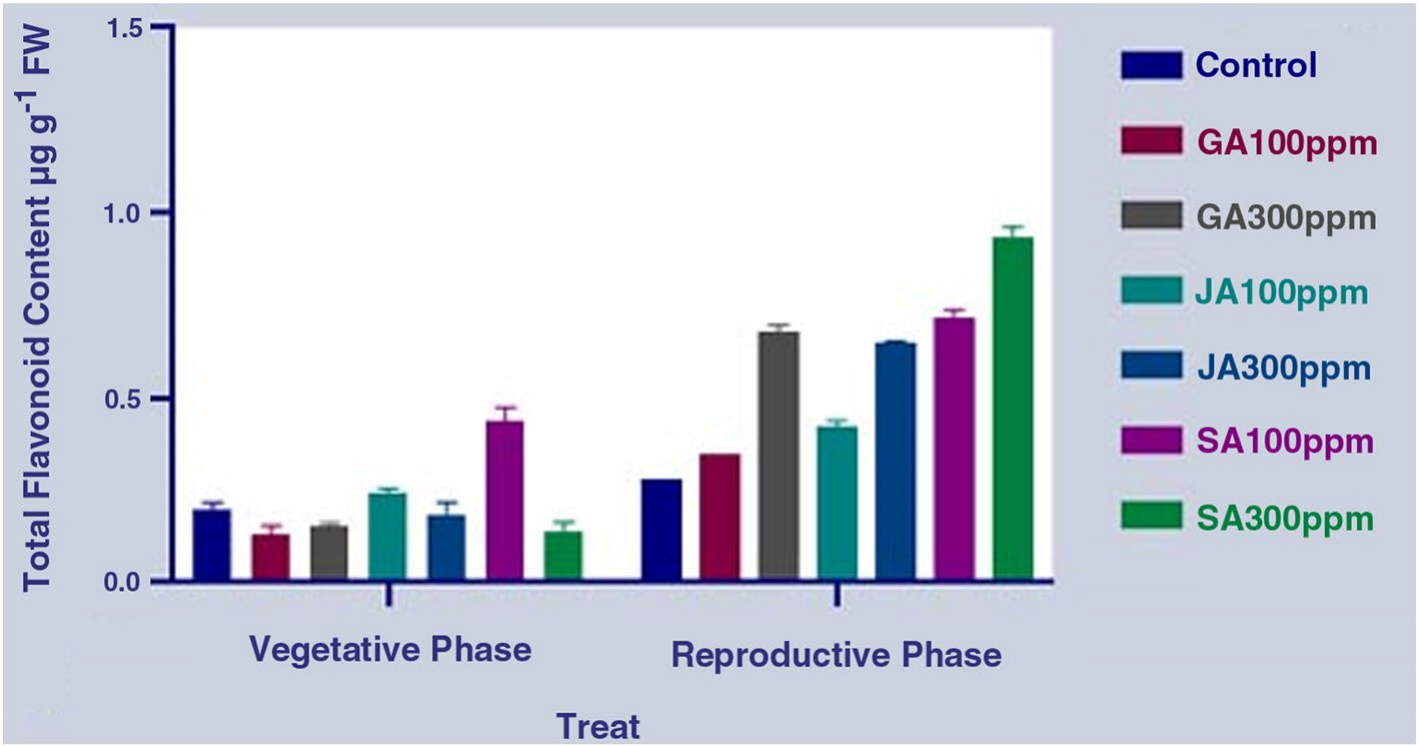
Diagram for comparing the effect of different hormonal treatments on the total flavonoid content in the aerial part at different phenological stages of *S. striata.*

**Figure 7:**
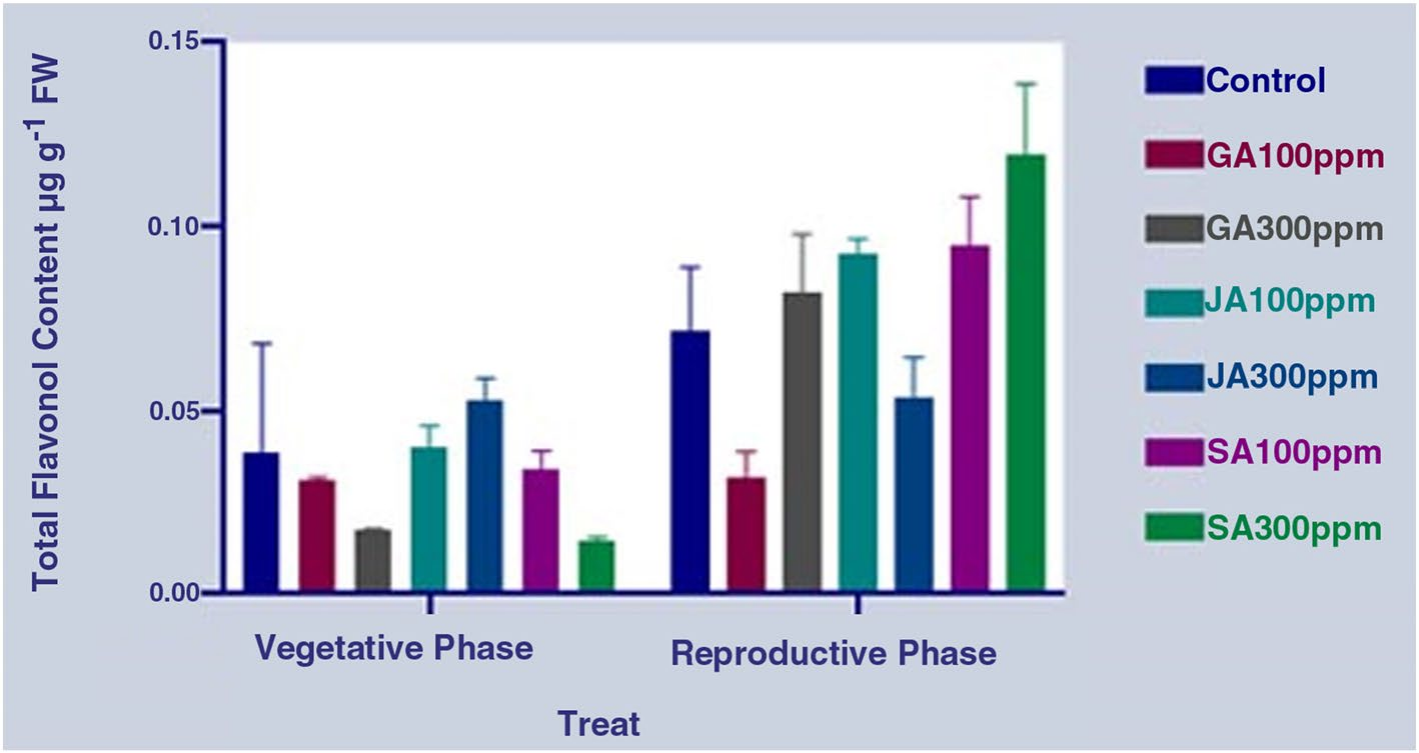
Diagram for comparing the effect of different hormonal treatments on the total flavonol content in the aerial part at different phenological stages of *S. striata*.

The results showed some significant changes in phenylpropanoid compounds such as phenol, flavonoids, and total flavonol contents under the influence of different concentrations of the hormones, including JA, SA, and GA, at different phenological stages in the aerial part of *S. striata* (P < 0.0001) (Table 3). The results of the comparison of the mean differences showed that the highest and lowest total phenol contents were obtained under the SA treatment (300 ppm) in the reproductive phase (50% of flowering) and under the JA treatment (300 ppm) in the same period, respectively. On the other hand, the results show no significant difference in the total phenol content during the reproductive period with the treatment with different concentrations of GA compared to the control treatment (Fig. 5). The results of measuring the flavonoid content in the aerial part also show the highest and the lowest flavonoid contents at the reproductive growth stage in the SA (300 ppm) concentration and GA (100 ppm) at the vegetative growth stage, respectively. In other words, the flavonoid content in the SA treatment (300 ppm) was about 2.5 times higher than that of the control treatment in the reproductive phase. Overall, the mean flavonoid content in the reproductive growth period was observed to be much higher than that of the vegetative phase (Fig. 6). The evaluation of the total flavonols in both control and treated cells of plant showed that the highest and lowest total flavonol contents were observed in the SA treatment (300 ppm) at the reproductive growth and the vegetative growth stages, respectively. Overall, it was found that the average flavonol content in the reproductive period was higher than the vegetative period. Additionally, there was no significant difference in terms of the flavonol content between the application of both SA and GA treatments and the control treatment in the vegetative phase (Fig. 7). Overall, by comparing the results of the measurement of phenylpropanoid compounds in the aerial parts of *S. striata* in the two vegetative and reproductive phases, it was revealed that the total phenol amount in the vegetative phase is 1.5 times higher than that of the reproductive phase. However, the flavonoid and total flavonol contents in the reproductive phase of the plant were 2.7 and 2.4 times higher than that of the vegetative phase, respectively. The application of SA treatment (300 ppm) in the reproductive phase caused a significant effect on the accumulation of all the three target metabolites.

**Table 3:** Analysis of variance of the effect of different hormonal treatments on the content of phenylpropanoid compounds in the aerial part at different phenological stages of *S. striata*.

S.O.V	DF	Total phenol	Flavonoid	Flavonol
Treat	6	****0.04811	****0.1066	***0.0009486
phenological stages	1	****0.2330	****1.364	****0.02162
Treat* phenological stages	6	****0.05294	****0.09151	****0.002063
Error	28	0.0002120	0.0004177	0.0001581
CV		4.31	5.18	22.69

### A correlation between the identified phenylpropanoid compounds and the CHS and C4H expressions at different phenological stages

The results of the correlation analysis show a very significant correlation between CHS gene expression and flavonoid content in the vegetative phase of the plant (0.83). As well, a positive and non-significant correlation was found between C4H gene and phenol and flavonoid contents at this stage. Moreover, there was a negative relationship between the expressions of both CHS and C4H target genes and flavonol content. On the other hand, a high and significant correlation was observed between CHS gene expression and flavonoid content in the reproductive phase, indicating the prominent role of CHS in the biosynthesis of flavonoid compound compared to phenol and flavonol contents. There was a positive and significant correlation between C4H gene and both flavonoid and flavonol contents in the reproductive phase. Furthermore, a positive correlation, which was not significant, was found between C4H gene expression and phenol content in the reproductive phase. The results of the correlation indicate that increasing the expression of the above-mentioned genes, which are among the key genes in the biosynthetic pathway of phenylpropanoid compounds in *S. striata*, can lead to an increase in the accumulation of the content of these compounds, especially in the flavonoid content. The results of correlation analysis using Pearson coefficient, which was performed to find the correlation between CHS and C4H genes, also revealed a positive correlation between these two genes, suggesting that the expression of other genes involved in biosynthesis pathway can be stimulated and increased during the biosynthesis process of phenylpropanoid compounds due to the induction of the C4H gene (Table 4).

**Table 4:** Analysis of correlation of the identified phenylpropanoid compounds.

	Coefficient analysis	CHS	C4H
Vegetative phase	Total Phenol	0.19	0.28
	Flavonoid	*0.83	0.44
	Flavonol	− 0.137	− 0.436
	CHS		0.36
	C4H		
Reproductive phase	Total Phenol	0.62	0.60
	Flavonoid	0.68*	*0.68
	Flavonol	0.16	*0.83
	CHS		0.34
	C4H		

## Discussion

Genetic manipulation for the effective induction of plant secondary metabolites is known as one of the most important methods that are currently used, in which the identification and genetic manipulation of the key genes in the biosynthesis of secondary metabolites like phenylpropanoid compounds, are of great economic values. The results of the present study show a significant difference in the profiles of phenylpropanoid compounds of different treatments. Elicitors have also been reported to induce physiological changes in plants. The external use of some compounds such as JA and SA, induces false stress in the plant and stimulates its defense responses, as well. In response to the oxidative stress, the plant increases the expression of antioxidant genes, thereby increasing the activities of both enzymatic and non-enzymatic antioxidants (often medicinal), which is consistent with the results of the present study^37,38^. Thus, the use of elicitors in limited quantities and at appropriate concentrations could stimulate or improve the biosynthesis of certain compounds in the living cell system and generally reduce the time of reaching high levels of metabolites^39^. On the other hand, considering the low expression of genes involved in the biosynthesis process of secondary metabolites, these elicitors can be used to increase the expression of those genes involved in their synthesis; therefore, the production of valuable metabolites in other plants and even in other organisms can be facilitated after being amplified and cloned in a suitable vector. Thus, it is important to select the appropriate elicitor for the production of large-scale secondary metabolites. The high expression of CHS gene in the arterial part of the plant in the reproductive phase in most of experimental treatments indicates that the synthesis of both phenolic and flavonoid compounds may be activated in these tissues, which then reaches its peak when the plant enters the reproductive phase. Similarly, in *Populus trichocarpa*, it was shown that PtrCHS4 was mainly expressed in leaves and stems and its expression was stimulated by wounding^40^. On the other hand, by comparing the phenylpropanoid compounds’ contents, a significant increase was observed in the flavonoid and flavonol contents in the reproductive phase in all treatments, especially the SA treatment (300 ppm). Accordingly, this is a reasonable result and CHS gene seems to play a more prominent role in the reproductive phase than in the vegetative phase. More precisely, downstream genes do not play a limiting role in the accumulation of both flavonoids and flavonols. However, the results of investigating the accumulation of total phenol content in the vegetative and reproductive phases indicate that a less prominent role of CHS for this compound belonged to the reproductive phase, because the total phenol content in the reproductive phase was lower than that of the vegetative phase. Therefore, it can be said that downstream genes play a more prominent role for the total phenol content in the reproductive phase. On the other hand, by comparing C4H gene expression levels in the arterial part in the vegetative and reproductive phases of the plant, it was shown that among the experimental treatments used, gene expression had a significant increase only at different concentrations of salicylic acid. In order words, this increase was more prominent in the reproductive phase than the vegetative phase and there was a decreasing or weak trend in the C4H gene expression in other treatments. After examining the accumulation of phenylpropanoid compounds, including flavonoids and flavonols, the effectiveness of this treatment (at different SA concentrations) and the prominent effect of C4H gene on their induction were confirmed. However, using other treatments where there was a decreasing trend in the gene expression and an increasing trend in the target metabolite accumulation, the role of downstream genes and the issue of precursor allocation applied by these downstream genes, were demonstrated. Overall, the results of CHS and C4H target gene expressions in *S. striata* arterial parts showed that SA treatment (300 ppm) was more effective on increasing the expressions of both genes and aerial parts in the reproductive phase in terms of the accumulation of target metabolites compared to other cases. Therefore, it can be concluded that the medicinal properties of plants vary according to their ages. In this regard, the bulk of medicinal plants at a certain age should be harvested in a certain season before being processed to produce the ultimate drug in order to prevent the changes in its potency. The results of the present study are inconsistent with previous reports, reporting that flavonoid content decreases during the plants’ growth period^41,42^. Wang et al. (2008) in their study stated that differences between mRNA and HDR protein levels in different organs may be due to post-transcriptional regulation and that the expression of HDR may mostly depend on the developmental stage^43^. Another point that may not completely and correctly reveal the results is the fact that the genes under study are regulated at the post-transcriptional level; therefore, the over- and under-expression of these genes cannot be definitively attributed to the variability in the levels of metabolites. In a study on three basil cultivars, researchers found a weak correlation between transcriptional level and enzymatic activities of TPSs, FDS, and GDS genes, which could indicate the role of post-transcriptional regulation^44^. Overall, the induction of bioactive secondary metabolites by chemical elicitors could enhance the free radical scavenging potentials of cell extracts. Flavonoids have antioxidant properties and it has been shown that their production as well as the expression of genes associated with their synthesis could consequently be increased under stress conditions^45^. A previous study indicated that the expression level of the CHS gene depends on the tissue and plant growth stage, and it is also affected by external stimuli such as UV rays, plant growth regulators, and drought^46,47^. Also, numerous reports have shown that SA increases the levels of some secondary metabolites, especially those involved in plant defense mechanisms^48^. Overall, elicitors were reported to have a positive effect on the transcription regulation of genes of vital enzymes in the phenylpropanoid pathway. For example, re-regulation of PAL transcripts has been previously reported by MeJa, SA, and SNP^49,50^, which are consistent with the results of the present study. Moreover, the elicitors have been reported to increase C4H gene expression as well as the biosynthesis of phenylpropanoid compounds. For example, in chickpeas, fungal elicitors could increase C4H gene transcription by 3.76 times^51^. Additionally, methyl jasmonate and methyl salicylate treatments in the rice leaf increased the C4H enzyme activity by 67% ^52^, which are consistent with the results of the present study. The positive correlation found between flavonoid content (catechin) and PAL and C4H expressions in tea indicates the relationship between phenylpropanoid and flavonoid pathways^53^. Therefore, to confirm the relationship between the expressions of CHS and C4H target genes and the accumulation of phenylpropanoid compounds in the *S. striata*, in the current study, correlation analysis was investigated between the genes expression and the phenol, flavonoids, and flavonol contents in the aerial part of the plant in both vegetative and reproductive phases. The results show that the highest positive and significant correlation belonged to CHS gene and flavonoid content in both vegetative and reproductive phases, which may indicate a more effective role of CHS gene on the biosynthesis of flavonoid content than other phenylpropanoid compounds in the aerial part. Furthermore, there was a high positive and significant correlation between C4H gene and flavonoid and flavonol contents in the reproductive phase. In a study conducted on *Carya illinoinensis*, the results of the correlation analysis between the content of phenolic compounds and the expression of CHS genes during the seed development period showed a significant relationship between changes in the expressions of CiCHS2 and CiCHS3 and changes in the total phenolic content. Therefore, it can be stated that CiCHS plays an important role in the synthesis of phenolic components in *Carya illinoinensis*^29^. Expressions of RgC4H and total phenol content in non-transgenic and transgenic plants in *R. glutinosa* indicated that this gene plays an important role in the biosynthesis of phenolic compounds. Indeed, overexpression or the decreased level of RgC4H gene in *R. glutinosa* changed tolerance to drought, salinity, and H_2_O_2_ stress^54^. The relationship between the phenolic accumulation and CHS expression in other species was also confirmed, which is consistent with the results of our report regarding the significant correlation between the content of phenylpropanoid compounds and the CHS gene. For example, a strong positive relationship was observed between flavonoid content and CHS expression in capillary roots of walnuts, induced by *Agrobacterium rhizogenesis* as well as a weak positive relationship between flavonol or proanthocyanidin content and CHS expression^55^. CoCHS gene is expressed in all tissues of *Coelogyne ovalis* especially in leaves and roots*.* As well, the expression of CoCHS increases at seedling stage under dark, cold, UV-B, wounding, and salinity stresses. A high correlation between CoCHS transcript expression and metabolite content such as flavonoid and anthocyanin, were observed at seedling stage under in vivo and in vitro conditions^28^. Accordingly, in another report, researchers demonstrated that the use of methyl jasmonate could induce the expression of CHS genes in citrus roots. On the other hand, in this study, there was a positive correlation between CitCHS expression and flavonoid production in the MeJA treatment^56^, which is consistent with the results of our report regarding the significance of the effect of non-biological elicitors on the accumulation of phenolic compounds through overexpression of target genes. Therefore, it is very likely that the CHS and C4H enzymes studied in the present study can consequently increase the production of phenylpropanoid compounds in the *S. striata*. In general, the first report of CHS and C4H gene expressions in *s. striata* showed that the use of different concentrations of hormone could be effective on gene expression, because this can increase the expression of key enzymes involved in the biosynthesis of phenylpropanoid compounds compared to the control. Indeed, the concentration of 300 ppm salicylic acid caused a significant increase in the expression of the CHS and C4H genes and the content of phenolic compounds.

## Materials and methods

### Plant materials and hormonal treatments

The studied seeds were collected from wild plants growing in the foothills of Ilam province located at Ilam University with a longitude of 46° and 28'​, latitude of 33° and 37', and altitude of 1174 m above the sea level and botanical characteristics of our samples were confirmed and also In coordination with the Gene Bank of Ilam University, plant sampling was performed according to the conventional methods of this gene bank, indeed all methods were carried out in accordance with institutional guidelines and regulations^57^. Thereafter, seed samples were collected with official permission obtained from Ilam University Gene Bank (permission letter), As well, some of the collected seeds are kept in the Gene Bank of Ilam University with code number IUGB02076. The seeds were firstly disinfected with 10% sodium hypochlorite and then washed with distilled water. In order to break the seeds’ coat dormancy, they were placed in a dark environment for 72 h at 24 °C with an optimal GA concentration (400 ppm). Thereafter, the treated seeds were disinfected and washed again with distilled water, placed on a filter paper in a petri dish, and finally transferred to a germinator. The germinated seeds were transferred to a research greenhouse after almost one week. Next, the factorial experiment was performed based on a completely randomized design (the first factor was the type of hormonal treatment, and the second factor was growth stage) in pots with an area of 0.15 m^2^ and equal ratios of soil, sand, and fertilizer. These pots were kept at daily temperatures of 25 °C and 20 °C, at a light intensity of 35,000 to 40,000 lx. Of note, the plants were watered daily. The experimental treatments included JA, SA, and GA (100 and 300 ppm) (prepared by Sigma Co.) along with the control treatment (distilled water). The seeds were sprayed three times with a 72 h interval at four-five leaf stage and then sampling was performed at two stages of vegetative phase and reproductive phase (50% flowering). Finally, the harvested leaf samples were stored in a freezer at − 80 °C for molecular and physiological studies.

### RNA and cDNA preparation

**Figure 8:**
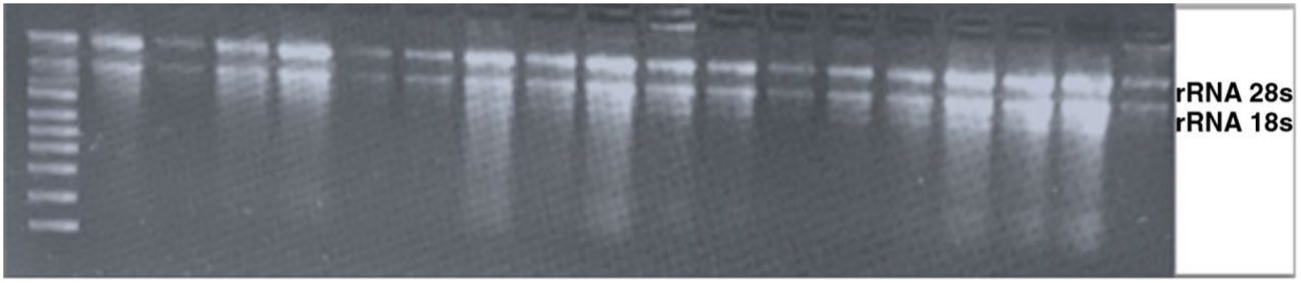
An example of the total RNA extracted of the *S. striata*.

RNA extraction was performed in terms of the instructions (George 2018) with some modifications^57^. In order to evaluate the quality of RNA, 1.2% agarose gel electrophoresis was used. Gel photography was performed by Gel Doc™ XR + device from BIORAD model and saved with Image Lab™ software. Thereafter, both the RNA quality and concentration were determined by calculating the adsorption ratio (A 260/280) and (A 260/230) using a NanoDrop device (NanoDrop1000 spectrophotometer) (Fig. 8). As well, total RNA (0.5 μg) was used to synthesize complementary DNA (cDNA) using thymidine oligomer (Oligo-dt18) as a primer. Synthesis of cDNA was performed using a kit (SMOBIO Taiwan) in terms of the manufacturer’s instructions.

### Isolation of CHS and C4H genes

**Figure 9:**
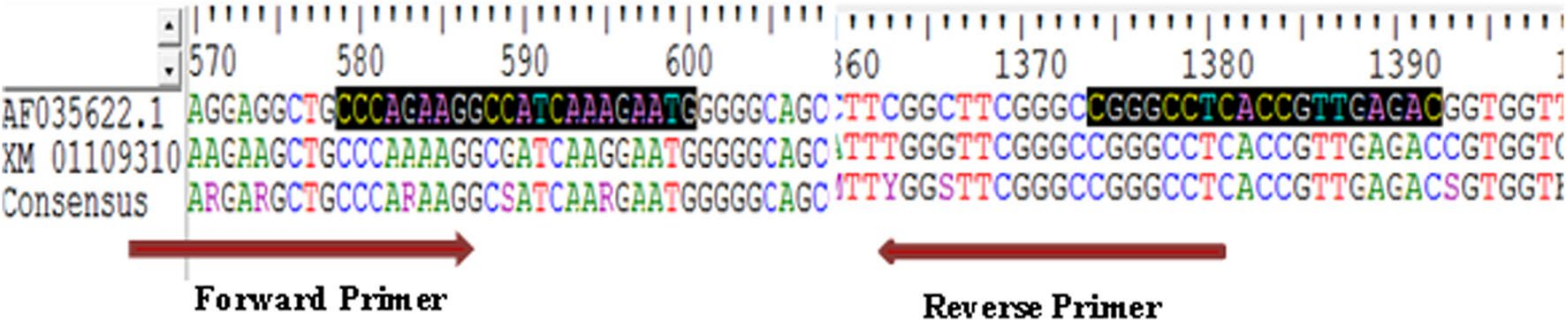
Results obtained from sequencing alignment by Clustalw method and primer design of protected regions in sequences used for the CHS gene.

**Figure 10:**
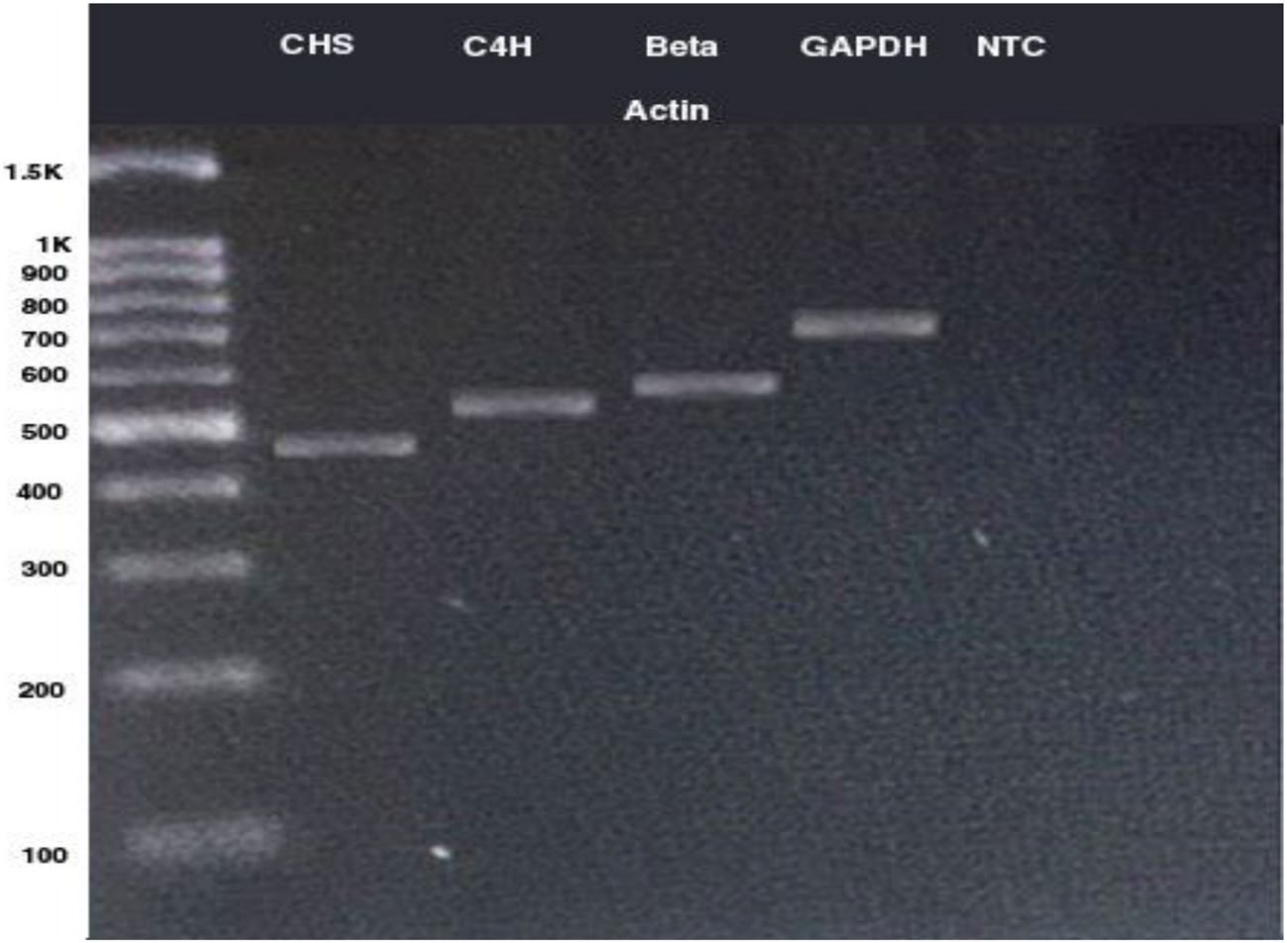
PCR analyses for CHS, C4H. Beta Actin and GAPDH genes.

To design the primers for the CHS and C4H genes, the NCBI and medicinal Plants -related databases were searched. As a result, the above-mentioned sequences for *S. striata* were not available in these databases, so the closest nucleotide sequences for the CHS and C4H were extracted from the NCBI website. Thus, based on the nucleotide searches in NCBI for plants of the lamiaceae family, *Scutellaria lateriflora* gene sequence with an access code (KF039680.1) and *Sesamum indicum* gene sequence with an access code (KP070827.1) were selected to design the C4H primer. Additionally, to design the CHS primer, the gene sequences of *Scutellaria baicalensis* with an access code (AF035622.1) and *Sesamum indicum* with an access code (XM_011093100.2) were selected. Thereafter, the sequences were extracted from both genes and then aligned in BioEdite Sequence Alignment Editor using ClustalW method. Subsequently, the conserved regions were identified in both samples and the primers were designed based on some characteristics such as melting temperature, G + C value, lack of dimer primer, and primer length for both forward and reverse primers using the http://biotools.nubic.northwestern.edu/OligoCalc.html. website. The agreed sequence was also determined to design the degenerate primer from the conserved regions based on AUPC codes of nucleotide sequences at https://www.bioinformatics.org/sms2/iupac using BioEdite Sequence Alignment Editor Software. To design the reverse primers, the region specified as the reverse primer, was placed at https://www.bioinformatics.org/sms/rev_comp.html, in order to obtain a reverse complement sequence, as shown in Fig. 2. Accordingly, this is as an example for CHS gene and this method was used for other genes, as well (Fig. 9, Table 5). In addition, the dimer and thermodynamic characteristics of the primers were investigated using Oligo calculator software. Eventually, the primers were lyophilized by Sinaclon Company using the HPSF (High Purity Salt Free) purification. Next, PCR was performed for each gene in a 25 μl reaction volume containing 2 μl of buffer (10X) PCR, 1.8 μl of 20 mM magnesium chloride, 1.8 μl of 1 mM dNTP, 0.5 μl of each one of the forward and reverse primers (10 picomol), 0.4 μl of Taq polymerase enzyme (5 units), 3 μl of undiluted cDNA, and 15 μl of double-distilled water. PCR was performed in Ependrof machine for five minutes in terms of the following schedule: initial denaturation at 94 °C, then 35 cycles including 30 s denaturation at 94 °C, 45 s of annealing each primer (at the recommended temperature to connect the primers), 1 min at 72 °C for both extension and amplification, and finally the final extension for 5 min at 72 °C. It should be noted that no-template control (NTC) or negative controls are used in PCR experiments. The resulted products were performed by horizontal electrophoresis and 1.8% agarose gel and 0.5 × TBE buffer at a constant voltage of 80 for 90 min. The gel was stained in ethidium bromide solution. Gel photography was performed by Gel Doc™ XR + device from BIORAD model and saved with Image Lab™ software (Fig. 10). To determine the nature and accuracy of the primers’ performance, PCR components were sequenced. Thereafter, to sequence the PCR product, it was placed in 1.5 L tubes and sealed with parafilm. Finally, the samples were sent on granular dry ice (− 70 °C) to Bioneer Corporation (South Korea) by Takapoozist Company. Finally, the sample purification was performed by Bioneer Corporation before sequencing process.

**Table 5:** Primers used in the study.

Gene	Primer	Primer sequence	Use
C4H	C4H-F	GGCGCCGTCRCTGGACGCC	Cloning
	C4H-R	GTCTCAACGGTGAGGCCCGGC	Cloning
	qPCR-F	GAAAGTGCACATATTCTATCCC	qRT-PCR
	qPCR-R	GATTTGGCATCTTTTCAAGGTAG	qRT-PCR
CHS	CHS-F	CCCARAAGGCSATCAARTGG	Cloning
	CHS-R	GTCTCAACGGTGAGGCCCG	Cloning
	qPCR-F	TGCCAGATGGAACGTATGG	qRT-PCR
	qPCR-R	ATGCCATCGAAGCCTGCTT	qRT-PCR
Bta actin	Beta actin-F	TCTGGAGATGGTGTGAGCCA	Cloning
	Beta actin-R	GGAAGGTACTGAGGG AGGCC	Cloning
	qPCR-F	CGGGATGGAAGCTGCTGG	qRT-PCR
	qPCR-R	CCGGTCAGCAATACCCGGG	qRT-PCR
	GAPDH-F	GATTACATGACMTAYATGTTCAAG	Cloning
GAPDH	GAPDH-R	ACATCATCYTCAGTGTAHCCC	Cloning
	qPCR-F	GACAAGGACAAGGCTGCTGC	qRT-PCR
	qPCR-R	ACACCAACAACAAACATGGGAGC	qRT-PCR

### Quantitative real-time PCR

C4H and CHS genes, which were treated with SA, JA and GA elicitors, each one at 100 and 300 ppm concentrations compared to the negative control (deionized water) at two stages of vegetative and reproductive growth of the *S. striata*, respectively, were partially amplified to measure gene expression by standard Real Time PCR. In this way, after the isolation and sequencing of some CHS and C4H genes, specific primers were designed to investigate these genes’ expression in *S. striata* (Table 5). Real Time PCR reactions were then performed in the final volume of 10 μL using RealQ Plus 2 × Master Mix Green no Rox kit in terms of the manufacturer's instructions (Danish Amplicon) by a CFX96 BIO-RAD device. In order to evaluate the expressions of CHS and C4H genes quantitatively in comparison with the internal control, the optimal concentration of the primers and cDNA was determined in the pilot experiments. Moreover, for each gene, the appropriate number of cycles and the optimal annealing temperature were determined using the cycle gradient and temperature. The best amplification conditions were determined as 40 cycles in the case of any failure in reaching a steady state in the PCR product amplification. Temperature conditions of Real Time PCR reaction include the initial activation of the enzyme for 10 min at 94 °C, 40 cycles consisting of denaturation for 5 s at 94 °C, annealing temperature (depending on the primer type 49–55) (Table 5) for 45 s, and extension for 1 min at 72 °C. In order to eliminate the fluctuations of RNA values in the reaction and errors of devices and individuals using this method, reference genes were used as the controls. In this experiment, we used two housekeeping genes, transcript of beta actin that is commonly used as a housekeeping gene standard in qPCR. In addition, GAPDH is one of the most common reference genes used to normalize the gene expression data and its expression is very constant^58–60^. After comparing the expression of the target gene with that of the internal control gene, determining the over- or under-expression of the target gene in the samples was made possible. Thereafter, the sequences obtained from the *S. striata*, Beta actin, and GAPDH genes were used as the reference genes to normalize the data. The relative quantity was then obtained by measuring the increase in fluorescence radiation resulted from binding to Syber Green dye. After performing the PCR-based amplification reaction, these raw data were extracted from the machine in the form of Ct (Threshold Cycle). After these reactions, the melting curve of each gene was analyzed. Finally, according to the resulted peaks, the specificity of other products and the absence of dimer primer were confirmed. It should be noted that no-template control (NTC) was used to test the genomic contamination in real-time PCR.

### Determination of total phenolic, flavonoid, and flavonol contents

To measure phenol, flavonoids, and total flavonols contents, methanolic extract was firstly extracted from the samples. Initially, 0.05 g of the leaf tissue was ground in liquid nitrogen containing 3 ml of 80% homogeneous methanol and then placed in water bath for three hours at 80 °C. Next, the sample was centrifuged at 5000 rpm for 20 min. Finally, the supernatant was reduced to 3 ml using methanol, and the resulted extract was used to measure the phenol, flavonoids, and flavonol contents. The total phenol content was measured using Folin–Ciocalteu method^61^. A total of 500 μl of folin–ciocalteu reagent was added to 50 μl of the methanolic extract. After 5 min, 500 μl of 7% sodium carbonate buffer was added to the obtained mixture. The samples were kept for 10 min at room temperature and finally the total phenol content was read at 765 nm using a spectrophotometer (UV–Visible model Cary-50 made by Varian Co.). Thereafter, the total phenol content was calculated in μg/gr/fresh weight^62^ using the standard glycolic acid curve. To measure the flavonoid content, 250 μl of 10% aluminum chloride buffer and 250 μl of 1 M potassium acetate buffer were both added to 1 ml of the methanolic extract, and the absorbance of the samples was then read at 415 nm using a spectrophotometer. Finally, the total flavonoid content was measured based on the standard quercetin curve^63^. In order to measure the flavonol content, 1 ml of 2% aluminum chloride buffer and 3 ml of 5% potassium acetate buffer were added to 1 ml of the methanolic extract, and the absorbance of the samples was read at 445 nm using a spectrophotometer. Finally, the total flavonol content was measured based on a standard routine curve^64^.

### Statistical analysis

Expression analysis of data was conducted in two and three technical and biological replications, respectively. After classifying the samples, 14 (2 harvest stages*7 treatment) treatment groups were created, all of which were compared with the control group. Typically, the two-way ANOVA was used to test for expression differences among different stages and the performed treatment methods. To confirm the results of two-way ANOVA test, its supplementary test, i.e. T-test, was used. Expression analysis was then conducted using ∆∆ct method^65^. All molecular analyses were performed using GenEx 6.1 software. Analysis of variance and figures of target metabolites (including phenol, flavonoid, and flavonol contents) was performed using the SAS 9.1 and Graph Pad Prism 8 (Graph Pad Prism Software, Inc. San Diego CA, USA). Furthermore, Pearson’s correlation coefficient was used to examine the association between the relative gene expression levels and phenylpropanoid compounds. The level of significance was set at 0.05.

## Supplementary Information

Supplementary Information 1.

Supplementary Information 2.

## Data Availability

All data generated or analysed during this study are included in this published article [and its supplementary information files S1 and S2].
