# Supplementary figures and images for: The isolation and expression analysis of cinnamate 4-hydroxylase and chalcone synthase genes of Scrophularia striata under different abiotic elicitors
Source: Sci Rep. 2022 May 17;12:8128. doi: 10.1038/s41598-022-12361-8 (PMC9114027; doi:10.1038/s41598-022-12361-8)

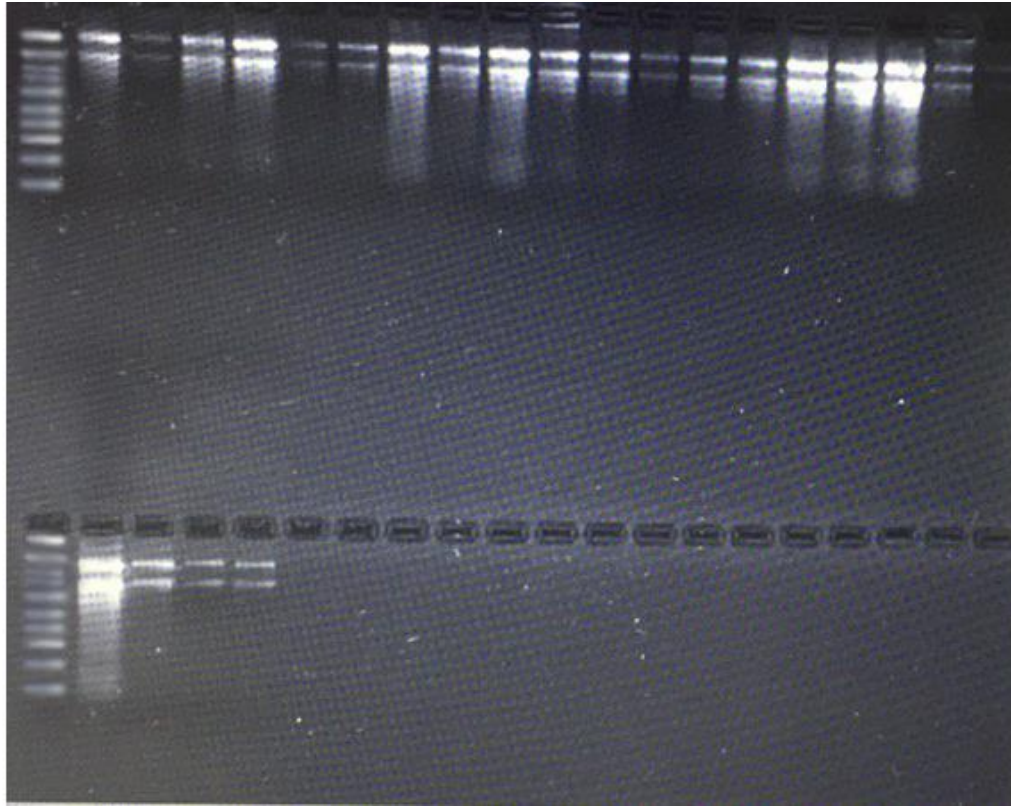

Figure 8. An example of the total RNA extracted of the *S. striata*.

Supplement: Supplementary file 1 — Supplementary Information 1. [file 41598_2022_12361_MOESM1_ESM.pdf]

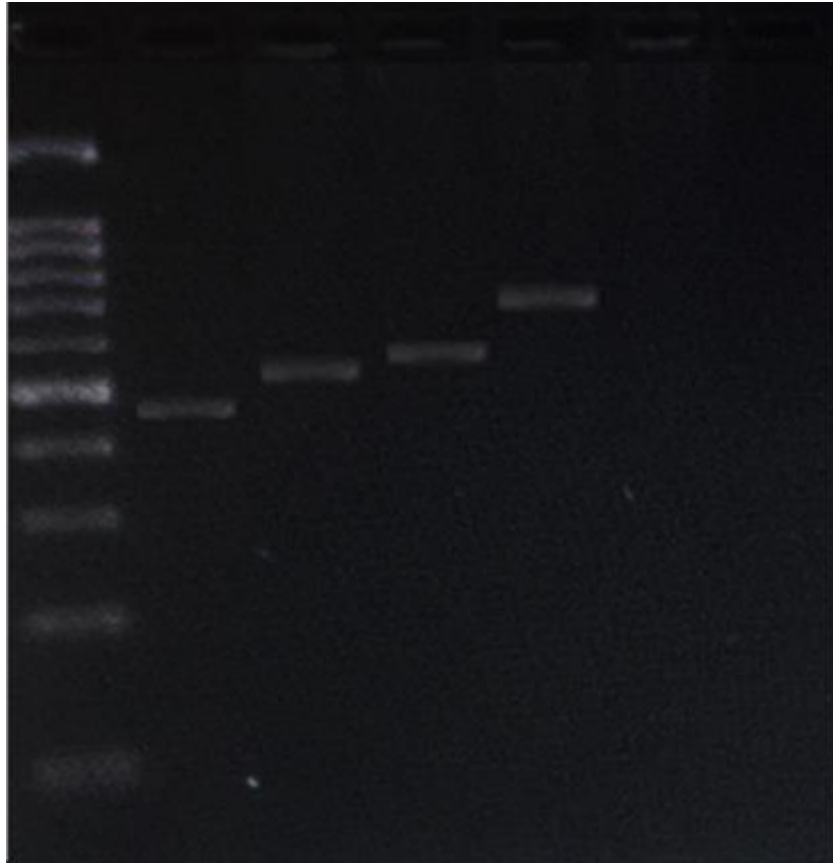

Figure 10. PCR analyses for CHS, C4H. Beta Actin and GAPDH genes and NTC sample.

Supplement: Supplementary file 2 — Supplementary Information 2. [file 41598_2022_12361_MOESM2_ESM.pdf]
